# Three odorant-binding proteins are involved in the behavioral response of *Sogatella furcifera* to rice plant volatiles

**DOI:** 10.7717/peerj.6576

**Published:** 2019-03-07

**Authors:** Kui Hu, Sheng Liu, Lin Qiu, Youzhi Li

**Affiliations:** 1 Hunan Provincial Key Laboratory for Biology and Control of Plant Diseases and Insect Pests, College of Plant Protection, Hunan Agricultural University, Changsha, Hunan Province, China; 2 National Research Center of Engineering & Technology for Utilization of Botanical Functional Ingredients, Hunan Agricultural University, Changsha, Hunan Province, China

**Keywords:** Odorant-binding protein, *Sogatella furcifera*, Fluorescence binding assay, Rice plant volatiles, Behavioral response

## Abstract

Plant volatiles play an important role in regulating insect behavior. Odorant binding proteins (OBPs) are involved in the first step of the olfactory signal transduction pathway and plant volatiles recognition. *Sogatella furcifera* is one of the most destructive pests of rice crops. Understanding the functions of *S. furcifera* OBPs (*Sfur*OBPs) in the host plant location and the behavioral responses of *S. furcifera* to rice plant volatiles could lead to improved, more environmentally-friendly, methods for controlling this pest. We found that *Sfur*OBP1 displayed only weak binding with all the tested volatiles. *Sfur*OBP2, *Sfur*OBP3 and *Sfur*OBP11 had different binding affinities to β-ionone. *Sfur*OBP2 and *Sfur*OBP11 had strong binding affinities to β-caryophyllene (K_i_ = 2.23 µM) and plant alcohol (K_i_ = 2.98 µM), respectively. The results of Y-olfactometer experiments indicate that *S. furcifera* was significantly repelled by octanal and n-octane but strongly attracted by (+)-limonene, acetophenone, 2-heptanone, n-hendecane, *α*-farnesene and β-ionone. Furthermore, the dsRNA-mediated gene silencing of *SfurOBP2*, *SfurOBP3* and *SfurOBP11* shifted the olfactory behavior of *S. furcifera* for β-ionone, α-farnesene and plant alcohol, respectively. These results suggest that the *Sfur*OBPs are involved in the recognition of rice plant volatiles, and several potential repellants and lures for controlling this pest.

## Introduction

The white-backed planthopper, *Sogatella furcifera* (Horváth), is a migratory pest in east and south-east Asia that damages rice crops by sucking phloem sap (Zhang et al., 2016). *S. furcifera* is also a vector of the southern rice black-streaked dwarf virus (SRBSDV), which was first discovered in Guangdong Province, China (Zhou et al., 2008). This disease has spread quickly to other parts to southern China and northern Vietnam where it continues to cause enormous damage to rice crops (Zhou et al., 2013). It is widely accepted that the best way to control disease is to control its vector, and that efficient identification and location of host plants is essential for the survival of phytophagous species. Therefore, a better understanding of how *S. furcifera* locates rice plants could lead to the development of more environmentally friendly management strategies for this economically significant pest.

The olfactory system of insects plays a crucial role in detecting chemical signals and mediates behaviors, such as mate choice, host identification, and oviposition (Sun et al., 2017; Yao et al., 2016; Zhu et al., 2016). Generally, biogenic volatile organic compounds (BVOCs) emitted by host plants are first captured by OBPs; small, water-soluble proteins of ∼120–150 amino acids that bind and deliver odorants through the aqueous sensillar lymph to receptors (Jacquinjoly & Merlin, 2004; Pelosi et al., 2006). The first insect OBPs were identified in the sensilla lymph of *Antheraea Polyphemus* (Vogt & Riddiford, 1981). There have now been decades of research including, gene identification, protein localization, ligand binding kinetics, RNA interference and crystal structural analysis, conducted to investigate how insect OBPs function in odor detection and olfactory signal transduction (Li et al., 2017; Sun et al., 2017; Wang et al., 2014a; Zhang et al., 2017; Zhu et al., 2016).

The function of OBPs in rice planthoppers has also been studied. Ten *OBPs* have been identified in *Nilaparvata lugens* (He et al., 2011; Zhou et al., 2014). Of these, *NlugOBP3* has been found to be involved in the identification and location of rice plants by nymphs (He et al., 2011). In *S. furcifera*, 12 *OBPs* have been identified, two of which, *SfurOBP2* and *SfurOBP11*, are significantly more highly expressed in the antennae. Although the ligand binding characteristics of these two OBPs have been detected (He & He, 2014), but only 18 rice plant volatiles included and there were no behavioral trials of *S. furcifera* to these rice plant volatiles. The function of *Sfur*OBPs in the host-plant determination is still not clearly. Therefore, it is extremely important and necessary to know more about the behavioral responses of *S. furcifera* to rice plant volatiles and the role of OBPs in host identification and location.

In this paper we present the results of experiments designed to determine the function of four *SfurOBP* genes; *SfurOBP2* and *SfurOBP11*, which are most highly expressed in the antennae (He & He, 2014) and *SfurOBP1* and *SfurOBP3*, which are most highly expressed in the abdomen were also studied (He & He, 2014), as a previous study has demonstrated that the abdomen-enriched *Nlug*OBP3 played an essential role in the rice plants location (He et al., 2011). The binding affinities of rice plant volatiles to these four *Sfur*OBPs were determined using fluorescence competitive binding assays *in vitro* and the behavioral responses of *S. furcifera* adults to rice plant volatiles were investigated using a Y-tube olfactometer. In addition, RNA interference (RNAi) was used to study the function of *Sfur*OBPs in the behavioral responses of *S. furcifera* adults for the volatiles which binding well to OBP and attract or repel to *S. furcifera*.

## Material and Methods

### Insect rearing, total RNA isolation and cDNA synthesis

*S. furcifera* were collected from rice fields in Changsha, Hunan Province, China, and reared in the laboratory with healthy rice plants at 26 ± 1 °C, 85% relative humidity (RH), under a 16-h photoperiod. Total RNA was isolated from the whole-body of *S. furcifera* adults using MiniBEST Universal RNA Extraction Kit (TaKaRa, Dalian, China) and first-strand cDNA was synthesized using the PrimeScript™ RT reagent Kit with gDNA Eraser (TaKaRa), following the manufacturer’s instructions.

### Expression and purification of recombinant proteins

The sequences of *SfurOBP1*, *SfurOBP2*, *SfurOBP3* and *SfurOBP11* were downloaded from NCBI GenBank (GenBank accession numbers KF732013, KF660218, KF732014 and KF732020, respectively). The *SfurOBP* genes were amplified by gene-specific primers (Table S1) and cloned into the vector pET-30a (+) using BamH I (TaKaRa) and Hind III (TaKaRa) restriction endonucleases. The pET-30a (+) vector allowed the expression of a recombinant product tagged with a His-tag sequence at the N-terminus. The recombinant plasmids were transformed into *Escherichia coli* DH5*α* competent cells (TaKaRa). The confirmed plasmids were transformed into *E. coli* BL21(DE3) cells (TaKaRa). The expression of recombinant proteins was induced with a final concentration of 0.4 mM isopropyl β-D-1-thiogalactopyranoside (IPTG). Recombinant proteins were purified by His-Tagged Protein Purification Kit (Cowin Biotech Co. Ltd., Beijing, China) and His-tag was removed using recombinant enterokinase (Novagen, Madison, WI, USA), according to the manufacturer’s protocols. Protein expression and purification were monitored by 15% SDS-PAGE. The concentration of highly purified proteins was determined by the standard bicinchoninic acid (BCA) method (Sangon Biotech Co. Ltd., Shanghai, China).

### Fluorescence competitive binding assay

Thirty-six rice odorants (He et al., 2014; Wang et al., 2017) and 1-N-phenyl-naphthylamine (1-NPN) were purchased from Sigma-Aldrich (St Louis, MO, USA). These odorants and 1-NPN were dissolved in high-performance liquid chromatography (HPLC) purify grade methanol for 1.0 mM as work solution.

Fluorescence competitive binding assays were performed on Tecan Spark 10M (Tecan Group Ltd., Männedorf, Switzerland) with the F96 Black ELIAS Plate (Sangon) (Khuhro et al., 2017; Liu et al., 2017). The solutions were excited at 337 nm and emission spectra were recorded between 390 and 490 nm. First, to test the binding constants of 1-NPN to the *Sfur*OBPs, a 2.0 µM solution of protein in 50 mM Tris-HCl (pH = 7.4) was titrated with 1 mM 1-NPN to achieve virous concentrations. Next, the competitive binding of each odorant was measured using the 1-NPN as fluorescent reporter and odorant as competitor. The concentrations of protein and 1-NPN were both 2.0 µM, the odorant added after protein and 1-NPN added into the well of ELIAS Plate for 2 min. The final concentrations of each competitor were 2, 4, 6, 8, 10, 12, 14, 16, 18 and 20 µM. After 2 min of odorant added, the fluorescence intensity was measured and recorded. The volume of mixed solution in each well was maintained at 250 µL. Each interaction was performed in triplicates.

GraphPad Prism 7.0 software (GraphPad Software, Inc., La Jolla, CA, USA) was used to calculated and generated the binding constants (K_1−NPN_) of 1-NPN to *Sfur*OBPs and relative Scatchard plots (Ban et al., 2003; Li et al., 2016). Dissociation constant (K_i_) of each odorant to *Sfur*OBP was computed from the corresponding IC_50_ value (the half maximal inhibitory concentration), using the following equation: K_i_=[IC_50_]/(1 + [1-NPN]/K_1−NPN_), where [1-NPN] is the free concentration of 1-NPN and K_1−NPN_ is the dissociation constant of the complex protein/1-NPN.

### Behavioral trials

To test the effect of the rice odorants on the behavior of *S. furcifera* adults, a glass Y-tube olfactometer (inner diameter 2.0 cm, stem 13 cm, arms 10 cm, angle of arms 60°) according to the previously described (Wang et al., 2014b) was employed. An airtight cubic box (70 by 45 by 30 cm) was used to position Y-tube, and the Y-tube was lighted by a 30-W filament lamp 25 cm above it. Air that filtered through activated charcoal and humidified with doubly distilled water was pumped in both arms at a flow rate of 300 mL/min. Two filter paper strips (10 by 1 cm) containing 100 µL odorant source (50 µL/L) and 100 µL control hexane were placed in two holding chambers in the front of the olfactometer, respectively. A *S. furcifera* adult (2 days after emergence, starved for 0.5 h) was randomly chosen and placed in the stem of the Y-tube. Each insect was given 10 min to choose between the two arms of the Y-tube, the choice was noted if the planthopper reached one-half of the arms’ lengths and stayed in the arm for more than 1 min. Besides, *S. furcifera* was considered as having no orientation preference. Forty-five individuals were used for each compound, each insect used only once. And the dual-choice experiment was done in an environmentally controlled room (25 ± 1 °C and 50% RH). A Chi-squared test was used to analyze the behavioral assay data by using SPSS19.0 (SPSS Inc., Chicago, IL, USA) software.

### RNA interference knock-down of *SfurOBP2*, *SfurOBP3* and *SfurOBP11*

*SfurOBP1* showed weakly binding affinity with all the tested volatiles, RNA interference experiments were employed in demonstrating the role of *Sfur*OBPs in rice plant volatiles perception. dsRNA for *SfurOBP2, SfurOBP3 and SfurOBP11* were synthesized and purified by using the T7 RiboMAX™ Express RNAi System (Promega, Madison, WI, USA) according to the manufacturer’s instruction. The *enhanced green fluorescent protein* gene (*EGFP*, GenBank accession No. U55762) was amplified as a negative control dsRNA (dsEGFP). The synthesized dsRNA was quantified by a spectrophotometer (NanoDrop™ 1000, Thermo Fisher Scientific, Wilmington, DE, USA) at 260 nm. The primers used to synthesize the dsRNA are listed in Table S1.

*S. furcifera* adults (one-day-old, CO_2_ anesthetized) were injected with dsRNA (50 nL/adult, 2,000 ng/µL) through the membrane between the meso- and meta-thoracic legs using a Nanoinjector (Drummond Scientific, Broomall, PA, USA). Five treatments including dsOBP2, dsOBP3, dsOBP11, dsEGFP and RNase-free water (Control) were set up. Each treatment consisted of 30 adults and the experiments were repeated three times. Ten individuals were collected from each replicate to determine the effects of RNAi by qPCR post 24 h of dsRNA injection (the timepoint with maximum RNAi efficiency after injection). Meanwhile, the behavioral responses of dsRNA treated adults for volatiles which binding well to OBP and attract or repel to *S. furcifera* were tested using a Y-tube olfactometer as described above.

### Real-time quantitative PCR

The expression levels of *SfurOBP2*, *SfurOBP3* and *SfurOBP11* were determined by qRT-PCR using a CFX96 Touch™ Real-Time PCR Detection Systems (Bio-Rad Laboratories, Hercules, CA, USA) and TB Green Premix Ex Taq™ II (TaKaRa) according to the manufacturer’s protocol. Three technical replicates were assessed for each biological replicate. The qPCR primers were designed using the National Center for Biotechnology Information profile server (http://www.ncbi.nlm.nih.gov/tools/primer-blast) and listed in Table S1. To calculate the relative expression levels, *α-1 tubulin* (*TUB*, accession No. KP735521) and *elongation factor 1-α* (*EF1-α*, accession No. KP735517) were used as the internal references according to the previous study (An, Hou & Liu, 2015). The data was analyzed using 2^−ΔΔ*Ct*^ method (Livak & Schmittgen, 2001).

## Results

### *In vitro* expression and purification of *Sfur*OBPs

**Figure 1:**
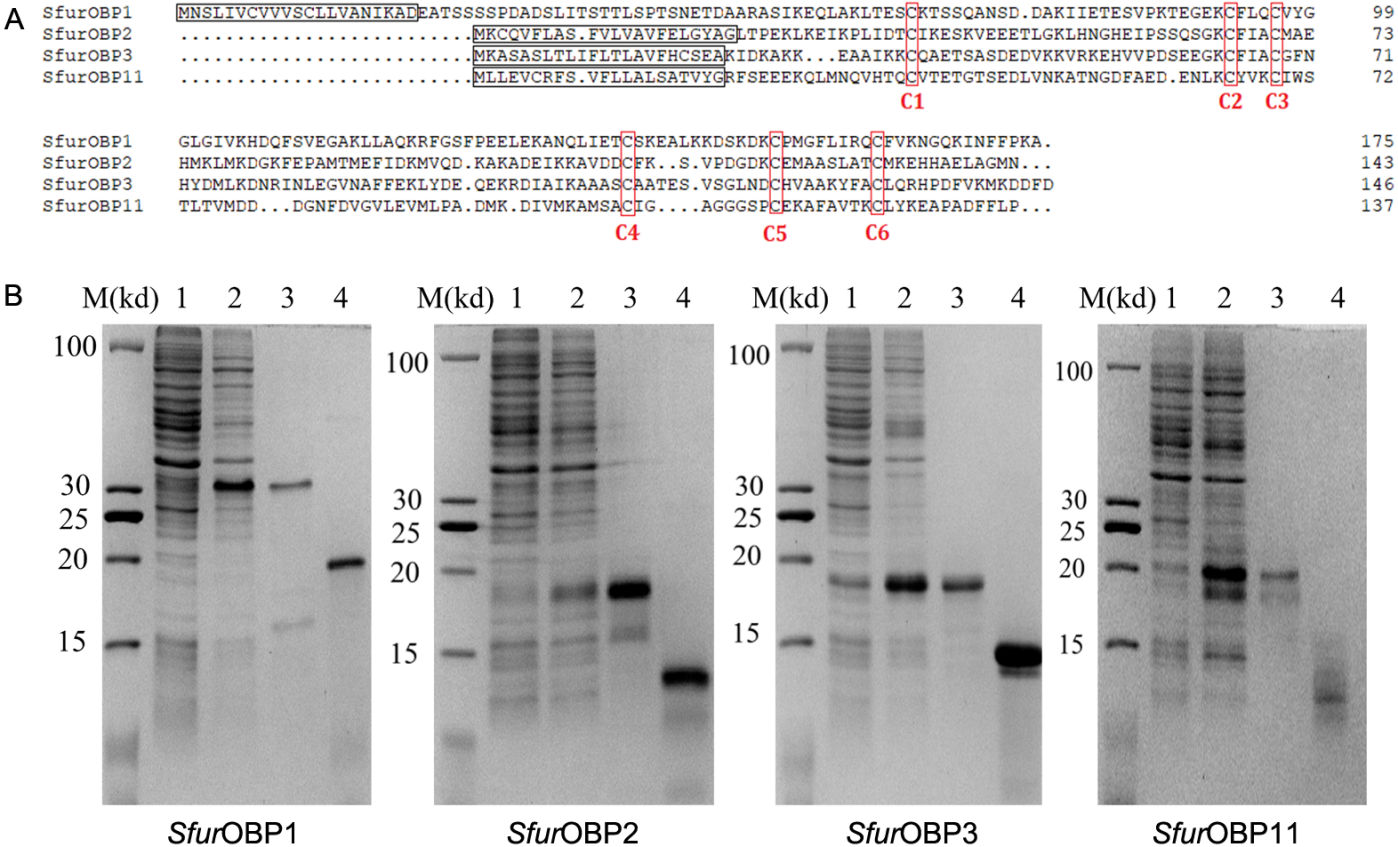
Sequence alignment of *Sfur*OBPs and production of recombinant *Sfur*OBPs. (A) Alignment of *Sfur*OBPs amino acid sequences. Predicted signal peptides are boxed, and conserved cysteines are labelled with red letter; (B) Expression and purification of the recombinant *Sfur*OBPs. M, molecular markers; 1 and 2, bacterial cells before and after induction by IPTG; 3 and 4, purified protein before and after cleavage by enterokinase.

*Sfur*OBP1, *Sfur*OBP2, *Sfur*OBP3 and *Sfur*OBP11 encode 175, 143, 147 and 137 amino acids, respectively, including six conserved cysteine residues. These four genes respectively contain 21, 22, 22 and 21 amino acids that encode predicted signal peptides (Fig. 1A).

The four *SfurOBP* genes were expressed in *E. coli* BL21(DE3), and recombinant *Sfur*OBP1, *Sfur*OBP2, *Sfur*OBP3 and *Sfur*OBP11 were produced as fusion proteins with a His-tag at the N-terminus which was, subsequently removed by treatment with enterokinase. The weights of purified *Sfur*OBP1, *Sfur*OBP2, *Sfur*OBP3 and *Sfur*OBP11 were predicted to be 17.50, 14.16, 14.70 and 13.34 kDa, respectively. Their purity and integrity were verified by sodium dodecyl sulphate polyacrylamide gel electrophoresis (SDS-PAGE; Fig. 1B).

### Fluorescence competitive binding assay

**Figure 2:**
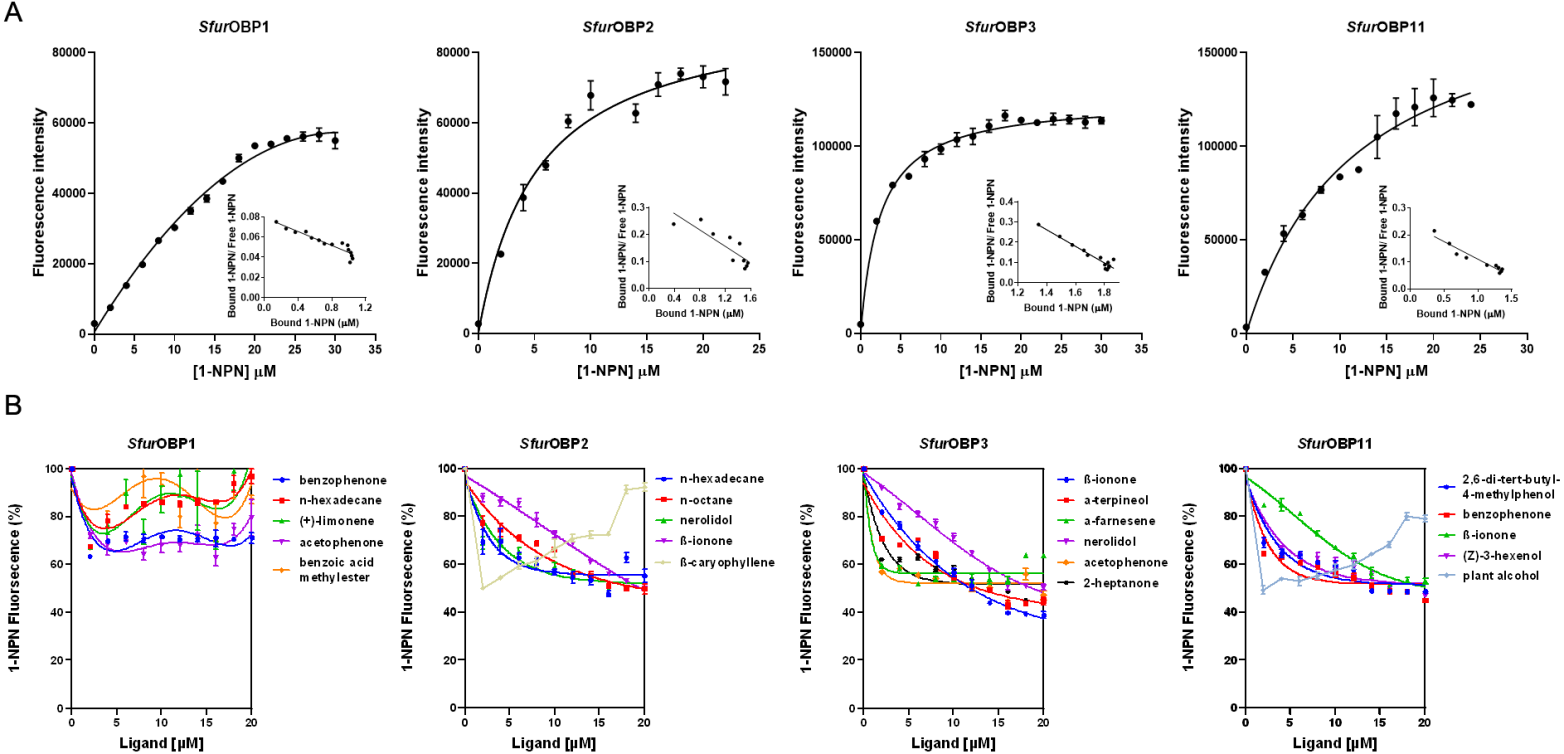
Binding curves of 1-NPN to SfurOBPs and binding curves of different ligands to *Sfur*OBPs. Bars indicate standard errors. (A) Binding curves of 1-NPN to *Sfur*OBPs and relative Scatchard plots of the four *Sfur*OBPs for 1-NPN (inset). (B) A few representative ligands are performed in the curves, the chemical name of tested ligands is shown on the right in the curves.

The dissociation constants of the fluorescence probe N-phenyl-1-naphthylamine (1-NPN) for purified *Sfur*OBP1, *Sfur*OBP2, *Sfur*OBP3 and *Sfur*OBP11 were 24.17, 5.67, 2.42 and 10.42 µM, respectively (Fig. 2A). A fluorescence competitive binding assay was used to determine the binding affinities of these four *Sfur*OBPs to 36 rice plant volatiles. The median inhibitory concentration (IC_50_) and dissociation constant (K_i_) values were calculated based on binding curves (Table S2). *Sfur*OBP1 displayed weak binding affinity (IC_50_ > 20 µM) to all 36 volatiles. *Sfur*OBP2 had moderate binding affinity to nerolidol, n-hexadecane, n-octane and β-ionone (K_i_ = 13.90, 13.31, 15.15 and 15.22 µM, respectively), and *Sfur*OBP2 strong binding affinity to β-caryophyllene (K_i_ = 2.23 µM) (Fig. 2B). *Sfur*OBP3 had high binding affinity to nerolidol, *α*-terpineol, 2-heptanone, acetophenone and β-ionone (K_i_ = 11.66, 8.38, 9.52, 10.67 and 7.37 µM, respectively) (Fig. 2B). *Sfur*OBP11 displayed moderate binding affinity to 4 volatiles; (Z)-3-hexenol, benzophenone, β-ionone and 2,6-di-tert-butyl-4-methylphenol (K_i_ = 16.72, 13.88, 16.91 and 15.35 µM, respectively). *Sfur*OBP11 had an especially strong binding affinity to plant alcohol (K_i_ = 2.98 µM) (Fig. 2B).

### Y-tube olfactometer assay

**Figure 3:**
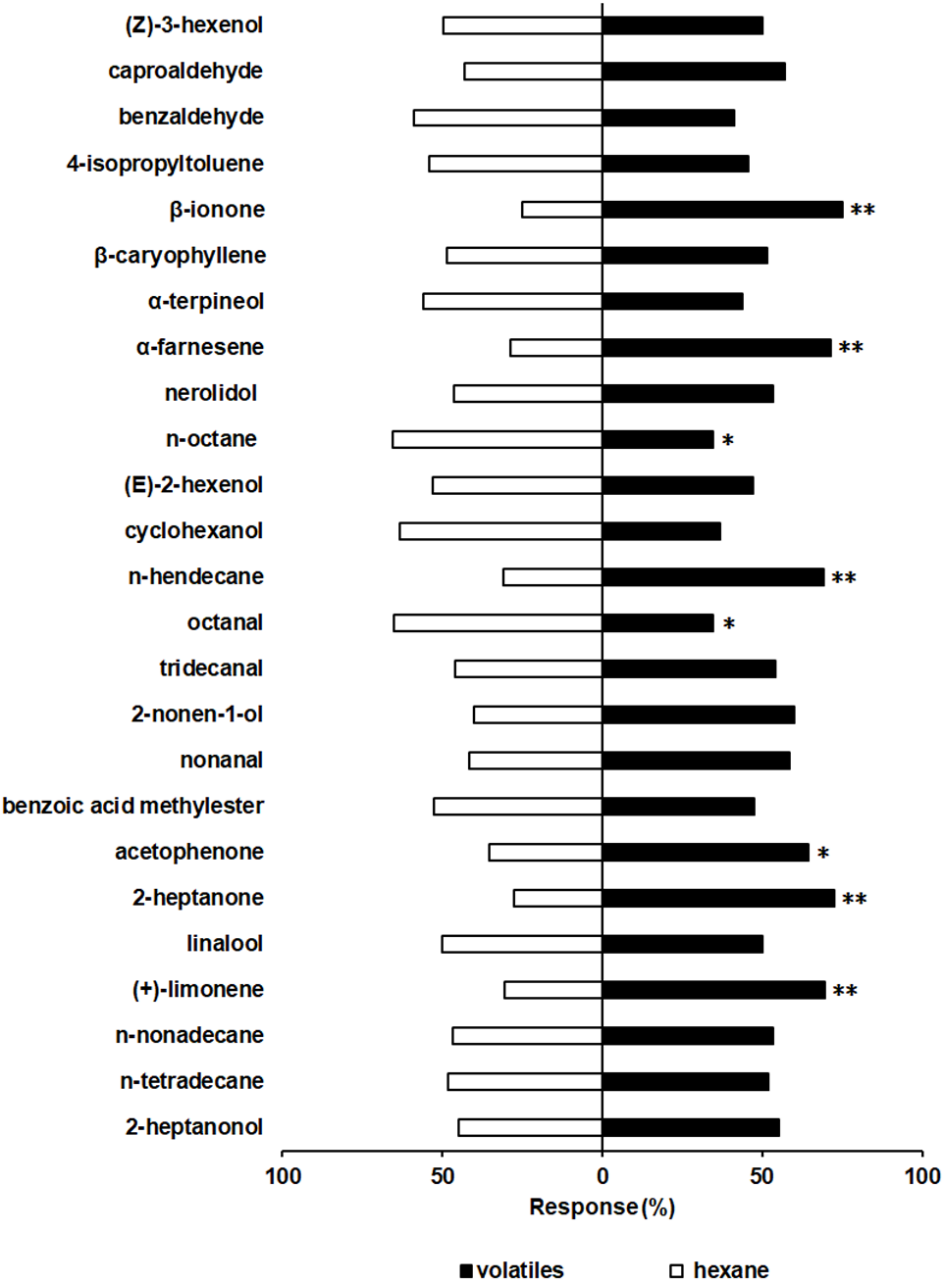
Behavioral responses of *S. furcifera.* in a Y-tube olfactometer bioassay when faced with the choice between different chemicals and hexane (Control). The difference of the insects choosing an odor was determined by a Chi-squared test, with the following levels of significance: *P < 0.05, **P < 0.01.

A Y-tube olfactometer was used to determine the behavioral responses of *S. furcifera* to the 36 rice plant volatiles. The behavioral responses of *S. furcifera* to 11 of these have been previous reported, including the significantly repellent effect of plant alcohol (Wang et al., 2017). The results of the present study show that *S. furcifera* is also significantly repelled by octanal (*χ*^2^ = 4.74, *P* < 0.05) and n-octane (*χ*^2^ = 4.94, *P* < 0.05) but is strongly attracted by (+)-limonene (*χ*^2^ = 7.96, *P* < 0.01), acetophenone (*χ*^2^ = 4.60, *P* < 0.05), 2-heptanone (*χ*^2^ = 10.58, *P* < 0.01), n-hendecane (*χ*^2^ = 7.68, *P* < 0.01), *α*-farnesene (*χ*^2^ = 9.23, *P* < 0.01) and β-ionone (*χ*^2^ = 13.33, *P* < 0.01) at the concentration of 50 µL/L (Fig. 3).

### Effect of RNAi treatment on the *SfurOBP* genes expression level and the behavioral responses of *S. furcifera* to the volatiles

**Figure 4:**
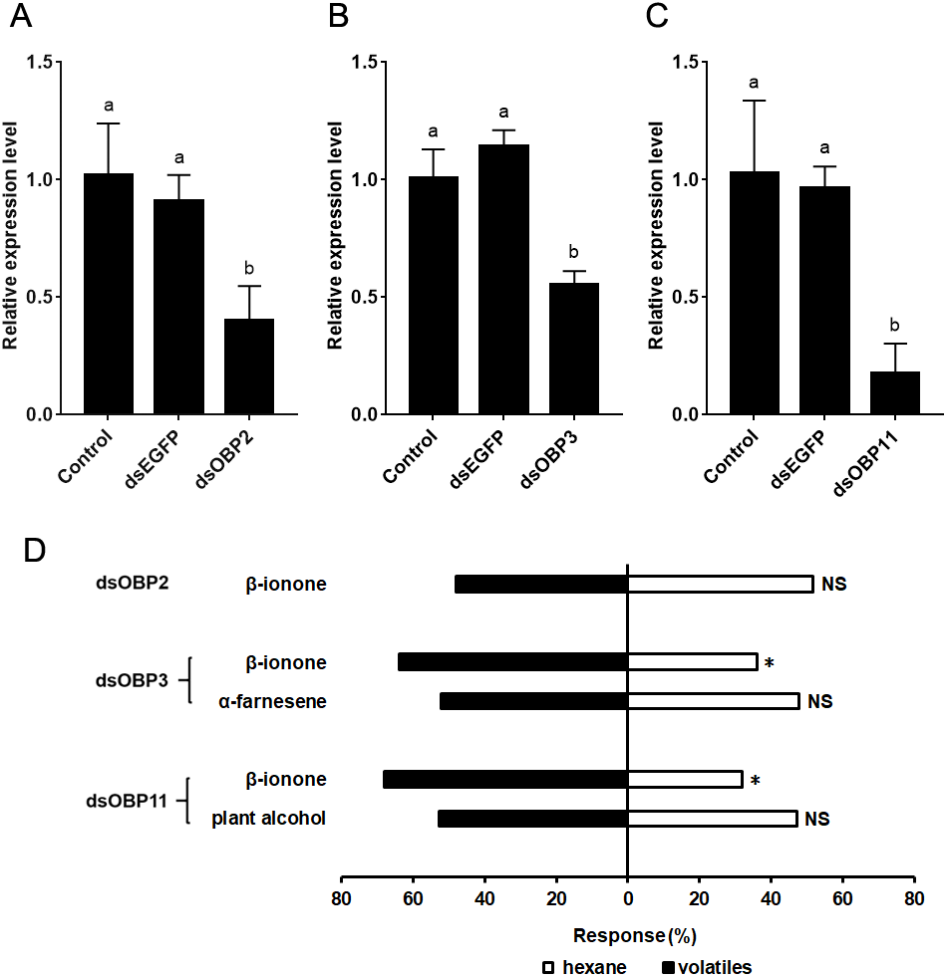
The relative expression levels of *SfurOBPs* in *S. furcifera* and behavioral responses of dsRNA treated *S. furcifera* in the Y-tube olfactometer bioassay. (A), (B) and (C) The mRNA levels of *SfurOBP2*, *SfurOBP3* and *SfurOBP11* in *S. furcifera* were measured at 24 h after injection of dsRNA. The bars represent mean ± SD of 3 biological replicates, different letters above bars indicate significant differences (one-way ANOVA, *P* < 0.05). (D) Behavioral responses of *S. furcufera* for rice plant volatiles. A Chi-squared test was used to determine the difference of the insects choosing an odor, with the following levels of significance: *P < 0.05. NS indicates no significant difference.

Twenty-four hours after dsRNA injection, the expression levels of *SfurOBP2*, *SfurOBP3* and *SfurOBP11* were significantly reduced by 60.0% (*F* = 12.284; *df* = 2, 6; *P* < 0.05) (Fig. 4A), 54.5% (*F* = 38.269; *df* = 2, 6; *P* < 0.05) (Fig. 4B) and 82.2% (*F* = 17.189; *df* = 2, 6; *P* < 0.05) (Fig. 4C), respectively, compared to control.

Silencing the *SfurOBP2* in *S. furcifera* significantly reduced the number of adults that attracted by β-ionone (*χ*^2^ = 0.080, *P* >  0.05, Fig. 4D). The *S. furcifera* no longer attracted by *α*-farnesene (*χ*^2^ = 0.080, *P* > 0.05, Fig. 4D) after injected with dsOBP3. Injection of dsOBP11 result in the plant alcohol lost the activation that repel to *S. furcifera* (*χ*^2^ = 0.180, *P* > 0.05, Fig. 4D).

## Discussion

Plant volatiles are important signal chemicals in insect-plant interactions (Allmann & Baldwin, 2010) and understanding the relationship between these chemicals and insect behavior can provide a theoretical basis for most sustainable pest control strategies.

In the present study, we investigated the ligand-binding properties of four *Sfur*OBPs and the behavioral responses of *S. furcifera* to rice plant volatiles. The results of the fluorescence binding assay show that *Sfur*OBP2, *Sfur*OBP3 and *Sfur*OBP11 all have binding affinity to β-ionone, a result that is consistent with other studies (Deng et al., 2012; Li et al., 2017; Venthur et al., 2016), *π*–*π* interactions between β-ionone and OBPs may be contribute to β-ionone’s ability to bind with different OBPs. Indeed, the finding that β-ionone binds strongly to *Hele*OBP can largely be attributed to *π*–*π* interactions between the ligand and Tyr113 in the binding site of *HeleOBP* (Venthur et al., 2016). This type of interaction had already been established when Tyr111 in the *Hobl*OBP1 binding was found to interact with β-ionone (Zhuang et al., 2014).

β-ionone is known to be a common fragrant odorant of rice (Fujii, Hori & Matsuda, 2010; Zhang et al., 2014), the results of our Y-olfactometer experiments show that *S. furcifera* is strongly attracted by this substance (50 µL/L). Furthermore, silencing the *SfurOBP2* of *S. furcifera* led the adults no longer attracted by β-ionone. These results suggest that β-ionone is involved in the host-plant recognition and location of *S. furcifera*. In fact, several previous studies have demonstrated that β-ionone is a potential bioactive volatile used by insect to identify and locate host plants (Venthur et al., 2016; Wei et al., 2013; Yang et al., 2015). In addition, our results also show that β-caryophyllene strongly bound to *Sfur*OBP2, which is highly expressed in the antennae (He & He, 2014). β-caryophyllene is a herbivore-induced rice volatile (Xu et al., 2002), and several previous studies have shown that it is strongly repellent to insects (Smith et al., 2012; Zhao et al., 2012). Consequently, we infer that similar concentrations of β-caryophyllene may repel *S. furcifera*. Furthermore, *SfurOBP2* is also highly expressed in the abdomen, it may be involved in some special physiological functions, such as immune response (He & He, 2014; Levy, Bulet & Ehretsabatier, 2004).

Our results indicate that nerolidol, *α*-terpineol, 2-heptanone, acetophenone, *α*-farnesene and β-ionone have high binding affinities to *Sfur*OBP3, and *S. furcifera* adults were attracted by 2-heptanone, acetophenone and β-ionone. In addition, injection of dsRNA-*SfurOBP3* affect the response of *S. furcifera* to *α*-farnesene (50 µL/L), *SfurOBP3* is indeed involved in this substance identification in *S. furcifera*. It is conceivable that conserved OBPs may have very similar functions. The ability of *N. lugens* nymphs to locate rice seedlings was significantly inhibited by silencing *NlugOBP3* with RNAi (He et al., 2011). Since, *SfurOBP3* shares 88% identity with *NlugOBP3* (He & He, 2014). We speculate that *Sfur*OBP3 is also involved in host plant identification and location in *S. furcifera*.

Plant alcohol, which is also strongly repellent to *S. furcifera* adult (Wang et al., 2017), also strongly bound to the other antennae-enriched OBP, *Sfur*OBP11. And silencing *SfurOBP11* dramatically decreased the number of *S. furcifera* repelled by plant alcohol (50 µL/L). Moreover, previous studies confirmed that RNAi-mediated gene silencing of *SfurOBP11* significantly reduced the ability of nymphs to find host plants (Jiang et al., 2016). Based on these findings, we infer that plant alcohol is mainly captured and transported by *Sfur*OBP11. Conversely, *Sfur*OBP1 had only weak binding affinity to all 36 tested volatiles (IC_50_ > 20 µM), which suggests that it is not involved in host plant identification, or at least not involved in identifying this particular group of host plant volatiles. Moreover, *SfurOBP1* is dominantly expressed in the abdomen, it may be involved in other physiological functions rather than olfactory functions (He & He, 2014).

In this study, we tested the behavioral response of *S. furcifera* for rice volatiles at the concentration of 50 µL/L. Certainly, it is possible that different behavioral response might be observed at other doses for the tested odorants, but further research is required. It’s possible that the plant volatiles either attract or repel *S. furcifera*, but don’t bind well to the four *Sfur*OBPs we tested, could be captured and transported by other *Sfur*OBPs. In addition, the β-caryophyllene and plant alcohol showed abnormal fluorescence binding curves (Fig. 2B), we observed an increase in fluorescence at high concentration of β-caryophyllene with *Sfur*OBP2 and plant alcohol with *Sfur*OBP11. These phenomenon may be due to the ligands might form micelles entrapping molecules of 1-NPN, they would produce a fluorescence peak in the same region of the spectrum as that relative to 1-NPN bound to the protein (Sun et al., 2012).

## Conclusion

In conclusion, our behavioral trails showed that eight compounds elicited significant behavioral responses from *S. furcifera*. Additionally, results of RNAi indicate that *SfurOBP2*, *SfurOBP3* and *SfurOBP11* are involved in the perception of rice plant volatiles in *S. furcifera*. Our researches will aid in developing environmentally friendly strategies to control this pest in the future.

##  Supplemental Information

10.7717/peerj.6576/supp-1 File S1 Primers used in this study

10.7717/peerj.6576/supp-2 File S2 Binding affinities of rice plant volatiles to *Sogatella furcifera* odorant-binding protein -1, -2, -3 and -11

10.7717/peerj.6576/supp-3 File S3 Raw data that applied for data analyses and preparation for Figs. 2–Fig. 4 and Table 2

## Additional Information and Declarations

### Data Availability

The following information was supplied regarding data availability: The raw date is available in File S3.
