# Supplementary material for: Three odorant-binding proteins are involved in the behavioral response of Sogatella furcifera to rice plant volatiles
Source: PeerJ. 2019 Mar 7;7:e6576. doi: 10.7717/peerj.6576 (PMC6409085; doi:10.7717/peerj.6576)
Supplement: File S1 [file peerj-07-6576-s001.docx]

**Table S1. Primers used in this study**

| **Purpose** | **Primer Name** | **Gene ID** | **Primer (5’→3’)** | **E (%)** | **R^2^** |
| --- | --- | --- | --- | --- | --- |
| Bacterial expression | *SfurOBP1*-F | KF732013 | AAGGATCCGACGAGGCGACGTCGTCATC^1^ | n.a. | n.a. |
|  | *SfurOBP1*-R |  | GGAAGCTTTTAGGCTTTGGGGAAGAAGTTTATC^2^ |  |  |
|  | *SfurOBP2*-F | KF660218 | AAGGATCCAGTCCGGCATTAACTGAAGC^1^ | n.a. | n.a. |
|  | *SfurOBP2*-R |  | GGAAGCTTTCAGAAGTTGAAGAAGAATGGATT^2^ |  |  |
|  | *SfurOBP3*-F | KF732014 | AAGGATCCAAAATTGACAAGGCCAAGAAGG^1^ | n.a. | n.a. |
|  | *SfurOBP3*-R |  | GGAAGCTTCTAAACATCGAAGTCGTCCTTCAT^2^ |  |  |
|  | *SfurOBP11*-F | KF732020 | AAGGATCCGGTCTCACCCCAGAAAAACTCA^1^ | n.a. | n.a. |
|  | *SfurOBP11*-R |  | GGAAGCTTTCAGTTCATACCAGCAAGCTCAG^2^ |  |  |
| qPCR | *SfurOBP2*-F |  | ATTCGAGCCAGCCATGACAA | 110.0 | 0.999 |
|  | *SfurOBP2*-R |  | TGAAGCAATCATCCACGGCT |  |  |
|  | *SfurOBP3*-F |  | GCCAAGAAGGAGGCCGCTAT | 107.7 | 0.969 |
|  | *SfurOBP3*-R |  | ATCCACAGGCGATGAAGCAC |  |  |
|  | *SfurOBP11*-F |  | CAGCGACAGTATATGGGCGA | 105.2 | 0.996 |
|  | *SfurOBP11*-R |  | GTCACCATTGGTCGCTTTGTT |  |  |
|  | *SfurTUB*-F | KP735521 | GAGGACACTACACCATCGGC | 99.3 | 0.992 |
|  | *SfurTUB*-R |  | TCAACAGCGAGGTGAATCCG |  |  |
|  | *EF1α-*F | KP735517 | AAGATCGGTTACAACCCGGC | 103.8 | 0.989 |
|  | *EF1α-*R |  | TCCTTGCGCTCAATGTTCCA |  |  |
| RNAi | *SfurOBP2*-F |  | TAATACGACTCACTATAGGGTCTCACCCCAAACTCAAAG^3^ | n.a. | n.a. |
|  | *SfurOBP2*-R |  | TAATACGACTCACTATAGGGGAAGTCACTTGGAGAAGCTCTG^3^ |  |  |
|  | *SfurOBP3*-F |  | TAATACGACTCACTATAGGGCAGCATCTCTTACCCTCATTTTTCT^3^ | n.a. | n.a. |
|  | *SfurOBP3*-R |  | TAATACGACTCACTATAGGGGCAATCGTTCAATCCACTTACAG^3^ |  |  |
|  | *SfurOBP11*-F |  | TAATACGACTCACTATAGGGTTATACCGGCAAGTGTGTGTTC^3^ | n.a. | n.a. |
|  | *SfurOBP11*-R |  | TAATACGACTCACTATAGGGGCTCAAGTCGGAATGTCTATCAC^3^ |  |  |
|  | *EGFP*-F | U55762 | TAATACGACTCACTATAGGGAGGACGACGGCAACTACAAG^3^ | n.a. | n.a. |
|  | *EGFP*-R |  | TAATACGACTCACTATAGGGGTCCATGCCGAGAGTGATCC^3^ |  |  |

^1^The underlined sequence indicate the position of the BamH I endonuclease site; ^2^The underlined sequence indicate the position of the Hind III endonuclease site; ^3^T7 RNA polymerase promoter is underlined; n.a. = not applied.
