# Supplementary material for: Three odorant-binding proteins are involved in the behavioral response of Sogatella furcifera to rice plant volatiles
Source: PeerJ. 2019 Mar 7;7:e6576. doi: 10.7717/peerj.6576 (PMC6409085; doi:10.7717/peerj.6576)
Supplement: File S2 [file peerj-07-6576-s002.docx]

**Table S2. Binding afﬁnities of rice plant volatiles to** ***Sogatella furcifera* odorant-binding protein -1, -2, -3 and -11**

| **Ligands** | ***Sfur*OBP1** | |  | ***Sfur*OBP2** | |  | ***Sfur*OBP3** | |  | ***Sfur*OBP11** | |
| --- | --- | --- | --- | --- | --- | --- | --- | --- | --- | --- | --- |
|  | **IC_50_ (μM)** | **K_i_ (μM)** |  | **IC_50_ (μM)** | **K_i_ (μM)** |  | **IC_50_ (****μM)** | **K_i_ (μM)** |  | **IC_50_ (μM)** | **K_i_ (μM)** |
| lauryl alcohol | >20 | - |  | >20 | - |  | >20 | - |  | >20 | - |
| cedrol | >20 | - |  | >20 | - |  | >20 | - |  | >20 | - |
| plant alcohol | >20 | - |  | >20 | - |  | >20 | - |  | 3.50 | 2.94 |
| linalool | >20 | - |  | >20 | - |  | >20 | - |  | >20 | - |
| cyclohexanol | >20 | - |  | >20 | - |  | >20 | - |  | >20 | - |
| (E)-2-hexenol | >20 | - |  | >20 | - |  | >20 | - |  | >20 | - |
| nerolidol | >20 | - |  | 18.21 | 13.46 |  | 18.98 | 10.40 |  | >20 | - |
| α-terpineol | >20 | - |  | >20 | - |  | 13.63 | 7.47 |  | >20 | - |
| 2-nonen-1-ol | >20 | - |  | >20 | - |  | >20 | - |  | >20 | - |
| (Z)-3-hexenol | >20 | - |  | >20 | - |  | >20 | - |  | 19.65 | 16.49 |
| 2-heptanonol | >20 | - |  | >20 | - |  | >20 | - |  | >20 | - |
| (+)-limonene | >20 | - |  | >20 | - |  | >20 | - |  | >20 | - |
| α-farnesene | >20 | - |  | >20 | - |  | >20 | - |  | >20 | - |
| β-caryophyllene | >20 | - |  | 2.93 | 2.16 |  | >20 | - |  | >20 | - |
| n-hexadecane | >20 | - |  | 17.44 | 12.89 |  | >20 | - |  | >20 | - |
| n-octadecane | >20 | - |  | >20 | - |  | >20 | - |  | >20 | - |
| n-tetradecane | >20 | - |  | >20 | - |  | >20 | - |  | >20 | - |
| n-nonadecane | >20 | - |  | >20 | - |  | >20 | - |  | >20 | - |
| n-hendecane | >20 | - |  | >20 | - |  | >20 | - |  | >20 | - |
| n-octane | >20 | - |  | 19.85 | 14.68 |  | >20 | - |  | >20 | - |
| 4-isopropyltoluene | >20 | - |  | >20 | - |  | >20 | - |  | >20 | - |
| naphthalene | >20 | - |  | >20 | - |  | >20 | - |  | >20 | - |
| methyl hexadecanoate | >20 | - |  | >20 | - |  | >20 | - |  | >20 | - |
| diisobutyl adipate | >20 | - |  | >20 | - |  | >20 | - |  | >20 | - |
| benzoic acid methylester | >20 | - |  | >20 | - |  | >20 | - |  | >20 | - |
| nonanal | >20 | - |  | >20 | - |  | >20 | - |  | >20 | - |
| tridecanal | >20 | - |  | >20 | - |  | >20 | - |  | >20 | - |
| octanal | >20 | - |  | >20 | - |  | >20 | - |  | >20 | - |
| benzaldehyde | >20 | - |  | >20 | - |  | >20 | - |  | >20 | - |
| caproaldehyde | >20 | - |  | >20 | - |  | >20 | - |  | >20 | - |
| benzophenone | >20 | - |  | >20 | - |  | >20 | - |  | 16.31 | 13.68 |
| 2-heptanone | >20 | - |  | >20 | - |  | 15.49 | 8.49 |  | >20 | - |
| acetophenone | >20 | - |  | >20 | - |  | 17.37 | 9.52 |  | >20 | - |
| β-Ionone | >20 | - |  | 19.95 | 14.75 |  | 11.99 | 6.57 |  | 19.87 | 16.67 |
| 2,6-di-tert-butyl-4-methylphenol | >20 | - |  | >20 | - |  | >20 | - |  | 18.04 | 15.14 |
| didecyl ether | >20 | - |  | >20 | - |  | >20 | - |  | >20 | - |

Ligand concentrations >20 μM for half-maximal relative ﬂuorescence intensity are represented as ‘-’
